# A comparison of educational strategies for the acquisition of nursing student’s performance and critical thinking: simulation-based training vs. integrated training (simulation and critical thinking strategies)

**DOI:** 10.1186/s12909-016-0812-0

**Published:** 2016-11-16

**Authors:** Nahid Zarifsanaiey, Mitra Amini, Farideh Saadat

**Affiliations:** 1 Department of E-learning, Virtual School, Center of Excellence for e-Learning in Medical Sciences, Shiraz University of Medical Sciences, Shiraz, Iran; 2 Quality improvement in clinical teaching Research Center, Education Development Center, Shiraz University of Medical Sciences, Sina- Sadra Halls Complex, Neshat Ave, Shiraz, Iran; 3 Fatemeh (P.B.U.H) College of Nursing and Midwifery, Shiraz University of Medical Sciences, Shiraz, Iran

**Keywords:** Simulation, Critical Thinking Strategies, Level of Performance, Nursing Students

## Abstract

**Background:**

There is a need to change the focus of nursing education from traditional teacher-centered training programs to student-centered active methods. The integration of the two active learning techniques will improve the effectiveness of training programs. The objective of this study is to compare the effects of the integrated training (simulation and critical thinking strategies) and simulation-based training on the performance level and critical thinking ability of nursing students.

**Methods:**

The present quasi-experimental study was performed in 2014 on 40 students who were studying practical nursing principles and skills course in the first half of the academic year in Shiraz University of Medical Sciences. Students were randomly divided into control (*n* = 20) and experimental (*n* = 20) groups.

After training students through simulation and integrated education (simulation and critical thinking strategies), the students' critical thinking ability and performance were evaluated via the use of California Critical Thinking Ability Questionnaire B (CCTST) and Objective Structured Clinical Examination (OSCE) comprising 10 stations, respectively. The external reliability of the California Critical Thinking questionnaire was reported by Case B.to be between 0.78 and 0.80 and the validity of OSCE was approved by 5 members of the faculty. Furthermore, by using Split Half method (the correlation between odd and even stations), the reliability of the test was approved with correlation coefficient of 0.66. Data were analyzed using *t*-test and Mann–Whitney test. A significance level of 0.05 was considered to be statistically significant.

**Results:**

The mean scores of the experimental group performance level were higher than the mean score of the control group performance level. This difference was statistically significant and students in the experimental group in OSCE stations had significantly higher performance than the control group (P <0.001). However, the mean scores obtained for the critical thinking did not increase before and after the intervention.

**Conclusion:**

The results showed that, the students’ performance level was increased by the application of integrated training (simulation and critical thinking strategies).

## Background

Technical and treatment advancements and rapid changes of political, social and cultural factors demonstrate the need to use new active training strategies in medical and nursing education. The nursing students should be actively trained in order for them to sufficiently understand real clinical situations and gain the learning in a real context [1, 2]. Deubel stated that the use of an educational theory and subsequent use of a teaching model alone is not sufficient to advance learning and in order to design active courses and create the ground for learners to think, educational spectrum and teaching models must be integrated. The integration of these practices such as simulation and critical thinking strategies will improve the effectiveness of training programs and will help students gain Nursing skills [3].

Students have interest in simulation as one of the forms of interactive learning as well as a powerful way of transferring skills, which can be performed individually or in small groups. There are many types of simulation such as live simulation, virtual simulation, structural simulation, role-playing simulation and use of mannequins [4, 5]. Simulation helps students to understand the importance of nursing interventions to patient outcomes by reflecting on the performance [6, 7]. It was revealed in different studies that simulation strengthens confidence, interest and clinical skills in nursing students [8]. Nursing students should be active learners and think critically to provide safe patient care. Hence, nursing education has stressed critical thinking as a necessary nursing skill [4].

Critical thinking is an essential component of professional responsibility and quality nursing care. In nursing, critical thinking is the ability to think systematically and reflect on the reasoning process used to ensure safe nursing practice [9]. Therefore, critical thinking skills are helpful in making appropriate decisions and quality nursing care. The development of critical thinking strategies and activities that facilitate this process will assist nurse instructors in planning appropriate educational strategies and assessment techniques. Simpson et al. believe that, critical thinking practices can change the students' focus from remembering to active learning [10]. Critical thinking strategies are the active learning strategies to promote critical thinking.

There are many educational methods for improving critical thinking such as using problem-based learning with case studies, group discussion and self reflection [11].

There is a close correlation between the simulation-based learning, critical thinking strategies and the principles of constructivist and collaborative learning. Furthermore, the integration of these strategies will strengthen learning [12], because students focus on problems in real clinical situations that could strengthen their understanding of concept and prepare them to manage complex clinical situations [6, 13]. One of the major problems in training the nursing students is the utilization of traditional and non-active education in the clinical training and practical skills, which makes it difficult for the students to follow the critical thinking, problem solving ability and clinical decision path [13, 14]. One of the problems for the nurse instructors is the implementation of critical thinking strategies in the curriculum and scenarios, which determine the clinical complications in making the right decisions for the patient [14].

In most reviewed studies, the integrated simulation training was investigated only with Problem based learning and the use of other critical thinking strategies (problem solving-based on group discussion) is less studied. The importance and necessity of this study is clear with respect to the special status of teaching the critical thinking skills, the sensitivity of teaching the clinical skills. In this way, we can empower nurses by adopting new techniques and strategies of active learning. Therefore, the aim of this study is to compare the effects of integrated training (simulation and critical thinking strategies) and simulation-based training on the performance level and the critical thinking ability of students in 2014 in Shiraz.

## Methods

### Study design

This research was a quasi-experimental research with non-equivalent group pretest posttest design, which was carried out to compare the effect of simulation-based training and integrated training (simulation and critical thinking strategies) on the performance level and the critical thinking ability of nursing students studying at Shiraz Faculty of Nursing and Midwifery in 2014.

### Samples and setting

The study samples include all first-year nursing students studying in the second semester of the academic year of 2014–2015 (*n* = 40) who were selected applying census method. In other words, the sample size is equal to the size of the population studied.

### Participants

#### Selection criteria

All students who took the 2-credit course of principles and practical nursing skills and wished to participate in the study were included.

Students who were enrolled as guest students at the semester and had no desire to participate in the research were excluded.

All the study subjects were divided into two groups according to age, sex, place of residence, and diploma average. Thereafter, two groups were randomly selected as the experimental group (*n* = 20) and the control group (*n* = 20).

##### Implementation stages of the research

1. Before the training, the level of critical thinking skills of both groups was investigated.
2. Two teaching techniques were carried out in each group. The experimental and intervention groups were trained using the integrated and simulation-based techniques, respectively.

##### Experimental group

The experimental group was trained for ten 2-h sessions using critical thinking strategies along with simulation.

The critical thinking strategies in this research include problem based learning with small group discussion

- At each session, hypothetical scenarios of clinical conditions related to the subject matter, prepared utilizing patient records in hospitals and reference books containing clinical setting and strategic questions, were discussed.
- Then, the solution to the problem was determined by students in small group discussion after changing the learning environment and the manner of seats arrangement in the lab in a U shape.
- At this stage, the instructor was used as a facilitator and provided students with the necessary instructions and tried to encourage all students to participate in the discussion.

**Fig. 1:**
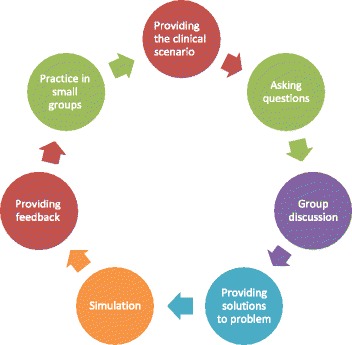
Integration cycle of the simulation and critical thinking strategies

2- Simulation stage (role-playing and mannequins):

- Then the students were taught the basic instruction by the instructor through role-playing, and they were also encouraged by the instructor to practice in groups of ten on the mannequins (Fig. 1).

##### Control group

The simulation method alone was used in the control group in such a way that the related instructor taught the subject matter of the same session on the mannequins through role-playing, without any clinical background and students practiced in groups of 10.

3. At the end of the program, both groups participated in the critical thinking skills test and Objective Structured Clinical Examination (OSCE) test in order to measure their performance. OSCE test was carried out by planning 10 stations.

### Data collection tool

The tool used in measuring the critical thinking ability was the California Critical Thinking Skills Test Form B (CCTST). This tool was developed to measure students' critical thinking skills and it contains 34 multiple-choice questions, designed in five areas of cognitive skills of critical thinking, which include analysis, inference, inductive reasoning, deductive reasoning and evaluation. Each person was awarded one score for each question which was answered correctly in the questionnaire and the total of correct answers constitutes the total score, the minimum and maximum of which were 0 and 34. Scores obtained in each section of the test was between the range of 0 and 16, such that in the analysis, inference, evaluation, inductive reasoning and deductive reasoning sections, 9, 11, 14, 16 and 14 scores were considered, respectively.

Objective Structured Clinical Examination (OSCE) test questions were utilized as a tool to assess the students' performance. This test was selected from 10 stations, including measuring blood pressure, dressing change, measuring body temperature, colostomy care, wound care clinical scenario, clinical bowel care scenario, pressure ulcer care, oxygen therapy, vital signs scenario, identifying devices by consulting with nursing team members.

#### Validity and reliability of tools

The external reliability of the California Critical Thinking Skills Test Form B(CCTST) was measured by Facione to be between 0.78 to 0.80 using the Kuder-Richardson formula 20 (KR-20) [15].

Objective Structured Clinical Examination (OSCE) validity was measured based on the content validity by five members of the faculty. Furthermore, the reliability of the test was approved with correlation coefficient of 0.66 using Split Half technique (the correlation between odd and even stations).

### Statistical analysis

Data were collected and imported into SPSS.V.15. Thereafter, they were analyzed and evaluated using descriptive and analytic statistics. The mean scores obtained from the performance level of the two groups were compared using the independent *t*-test, after the intervention. The pair *t*-test was utilized to compare the mean scores for critical thinking before and after the intervention. In addition, Mann–Whitney test and chi-square test were utilized to compare different fields of the critical thinking.

A significant level of P < 0.05 was considered to be statistically significant.

### Study challenges

One of the major limitations of this study was the presence of both groups of students in a collaborative learning environment. In addition, the implementation time of the training program of both groups was different due to less interaction between the two groups. Another limitation of this study was lack of educational sessions. Moreover, there was no possibility to hold more sessions in the curriculum of the faculty as a result of the coincidence of class hours.

## Results

Among the 40 first-semester nursing students who participated in the study, 25 were females (62.5%) while 15 were males (37.5%). There was a total of 13 female (65%) and 7 male students (35%) in the experiment. Moreover, there was a total of 12 female (60%) and 8 male students (40%) in the control group. The "Chi- square" test showed that the subjects in both experimental and control groups were homogenous in terms of sex. The mean age in the experimental group (19.75 ± 1.16) and in the control group (16.70 ± 1.16) was not significantly different (*p* =0.909). Furthermore, the average GPA in the experimental group (18.14 ± 1.22) and in the control group (17.87 ± 1.80) was not significantly different (*P* =0.597). In addition, there were 15 students (75%) and 5 students (25%) in the experimental group who were respectively living on and off campus. Moreover, in the control group, there were 17 students (85%) and 3 students (15%) who were living on and off campus. Using "Chi square" test, the frequency distribution of accommodation shows that there is homogeneity between the two groups.

The first hypothesis of the research states that "the integrated training (simulation and critical thinking strategies), compared with simulation training, improves students' performance." For this reason, the mean and standard deviation of the critical thinking score was evaluated in experimental and control groups before and after the intervention (Table 1).

**Table 1:** Comparison of mean and standard deviation of critical thinking score in the experimental and control groups before and after intervention

Critical Thinking Score	Mean and Standard Deviation		*T* test	Df	Sig
Educational groups	Before Intervention	After Intervention			
Experimental group	11.10 ± 3.47	10.95 ± 3.15	0.206	38	0.838
Control group	9.5 ± 2.56	9.10 ± 3.27	0.206	36.08	0.838

As shown in Table 1, there was no increase in the average scores of the critical thinking sub-groups before and after the intervention and the use of both the integrated strategies and the simulation-based training (critical thinking and simulation) methods alone didn't improve the critical thinking ability. Furthermore, the critical thinking sub-groups were studied in two groups before (Table 2) and after the intervention (Table 3).

**Table 2:** Comparison of mean and standard deviation of critical thinking subgroups scores in both groups before the intervention

Educational groups	Mean and Standard Deviation		*T* test	Df	Sig
Critical Thinking Score	Before Intervention	After Intervention			
Analysis	3.20 ± 1.39	2.90 ± 1.02	0.775	38	0.509
Evaluation	4.15 ± 1.89	3.10 ± 1.80	1.793	38	0.589
Inference	3.75 ± 1.80	3.50 ± 1.46	0.481	38	0.721
Deductive reasoning	5.80 ± 2.33	5.60 ± 2.08	0.286	38	0.223
Inductive reasoning	3.95 ± 2.50	3.00 ± 2.1	1.300	38	0.803

**Table 3:** Comparison between the mean and standard deviation of critical thinking sub-group scores in the training groups after the intervention

Educational groups	Mean and Standard Deviation		*T* test	Df	Sig
Critical Thinking Score	Before Intervention	After Intervention			
Analysis	2.8 ± 1.87	1.85 ± 1.56	1.884	38	0.228
Evaluation	4.05 ± 2.25	4.15 ± 1.53	−0.164	38	0.694
Inference	4.05 ± 1.43	3.10 ± 1.73	1.903	38	0.622
Deductive reasoning	5.75 ± 2.02	5.30 ± 2.12	0.685	38	0.753
Inductive reasoning	4 ± 1.39	3.50 ± 2.10	0.893	38	0.860

It was shown in Table 3 that, the average scores of the critical thinking in sub-groups of evaluation, inference, deductive and inductive reasoning were not statistically significant after the intervention in both experimental and control groups. However, Mann–Whitney test showed that there was a significant difference between the groups in the analysis dimension after the intervention (Table 4).

**Table 4:** Mean and standard deviation of critical thinking score in the analysis dimension in the experimental and control groups after the intervention

Critical Thinking Score in the analysis dimension	Mean and Standard Deviation	Mann–Whitney test	Z	Sig
Educational Groups				
Experimental group	2.85 ± 1.78	117	−2.33	0.020
Control group	1.85 ± 1.56			

As can be seen in Table 4, the Mann–Whitney test showed that the mean score of the critical thinking in the analysis dimension in the experimental group was higher than the mean score of the critical thinking in the analysis dimension in the control group, after the intervention, which was statistically significant.

The second hypothesis states that, the "integrated training (simulation and critical thinking strategies) compared with the simulation-based training improves students' performance." The mean and standard deviation of scores in OSCE (the performance level) were compared in 10 stations in both groups (Table 5).

**Table 5:** Comparison between the mean and standard deviation of scores obtained from OSCE test (the performance level) in 10 stations in the training groups

Educational groups	Mean and Standard Deviation		*T* test	Df	Sig
Evaluation station	Before Intervention	After Intervention			
1 Measuring blood pressure	9.28 ± 0.88	8.80 ± 0.797	0.775	38	0.079
2 Dressing change	8.97 ± 0.865	8.15 ± 1.40	1.793	38	0.031
3 Measuring body temperature	8.25 ± 1.99	8.25 ± 2.41	0.481	38	0.972
4 Colostomy care	8.27 ± 1.03	8.24 ± 0.554	0.286	38	0.257
5 Wound care clinical scenario	7.95 ± 1.60	8.65 ± 1.26	1.300	38	0.134
6- Clinical bowel care scenario	7.57 ± 1.97	5.79 ± 1.75	3.016	38	0.005
7- Pressure ulcer care	9.87 ± 0.55	8.68 ± 1.83	2.769	38	0.052
8- Oxygen therapy	8.31 ± 1.42	7.56 ± 1.48	1.630	38	0.111
9- Vital signs scenario	7.99 ± 1.85	5.29 2.48	3.890	38	<0.001
10- Identifying devices	9.37 ± 1.59	8.37 ± 3.03	1.731	38	0.086

Total scores were obtained in OSCE test which consisted of 10 stations with 10 scores. In stations 1, 2, 4, 6, 7, 8, 9 and 10, the average scores of the experimental group were higher than the average scores of the control group, where the difference was statistically significant at three stations of 2, 6 and 9. The scores obtained at stations 7 and 10 that were not normally distributed, were analyzed using non-parametric Mann–Whitney test. Here, the station 7 was statistically significant (p = 0.052). The average scores of both groups were equal at station 3. Furthermore, at station 5, the average scores of the experimental group were higher than the control group, which was not statistically significant. Generally, the average scores of the performance level of the experimental group were higher than the control group, which was statistically significant (Table 6).

**Table 6:** Comparison between the mean and standard deviation of scores obtained in OSCE test (the performance level) in both groups

The performance level score	Mean and Standard Deviation	Sig
Educational groups		
Experimental group	87.35 ± 6.20	0.0001
Control group	79.9 ± 7.35	

In total, the results showed that the experimental group obtained higher performance score, with statistically significant difference, than the control group (*p* = 0.001).

## Discussion

The aim of this study was to compare the effects of the integrated training (simulation and critical thinking strategies) and simulation-based training on the performance level and critical thinking ability of nursing students. The results of this study showed that the performance level of students in the experimental group (the integrated training) was higher than the performance level of students in the control group (simulation-based training) (*p* <0.001). Also, the results showed that the score of the critical thinking ability obtained in the integrated training was increased only in the analysis dimension. In other words, this training method didn't fully strengthen students' critical thinking ability. This finding is in line with similar results regarding the effects of the simulated patient (high-fidelity) [16, 17], effect of lecture method alone, lecture and case study, and the simulated patient [14] on critical thinking of nursing students. The results showed that, the critical thinking scores were improved, but there were no significant difference between groups. In summary, it can be concluded that a training course alone is not significantly correlated with the critical thinking. Furthermore, acquiring the critical thinking skills needs long period of time and continuing education. Moreover, the results showed that there was a significant relationship between the score of the performance level of the group which received simulation-based training alone and the one, which received the integrated training (simulation and critical thinking strategy) (*P* < 0.001). The result shows that integrated use of the training methods leads to higher performance in students. Previous studies have also revealed similar results regarding the positive effect of using integrated methods on clinical practice, problem solving skill, clinical efficacy and academic achievement and clinical competency. Moreover, in a study carried out by Liw et al. (2010) on first year nursing students in Singapore, which was conducted using the integrated training and problem- based training, the performance score of students who participated in the integrated education was higher than the performance score of those who received the problem- based training alone [18]. Lee et al. (2009) in Korea investigated the effectiveness of integrating two training methods (critical thinking strategies with simulation) on the empowerment in nursing fundamentals. The results suggested that the problem-solving skills and self-directed learning was significantly improved in the experimental group. Thus, the integrated method was proposed as a useful strategy in nursing education [19].

This study showed that the integration of critical thinking strategies (problem based learning with small group discussion) with simulation can improve the practical learning. The integration of both active learning will provide the opportunity to practice clinical skills in a real non-threatening environment [20, 21]. This helps students to have personal interpretation from problems and therefore strengthens the learning processes and problem-solving skill [22, 23]. In this research, students were given the opportunities to deeply discuss and explore the scenario through the integration of simulation and critical thinking strategies. The theoretical knowledge obtained from the scenario analysis facilitates the transfer of theoretical knowledge to students' clinical performance during simulation as well as encourages the discussion after simulation synthesis and application of the knowledge.

## Conclusion

The findings of the present study suggest that the use of integrated training methods (simulations and critical thinking strategies) as an active learning and student-directed strategies increase students' practical learning in a safe and controlled environment. This participatory approach is a deep learning guide for the learner in order to think aloud and explore the knowledge, solve problems and think critically.

Group dynamics on the development of critical debate and immersion in the simulation cycle enable students to use various sources to understand the functions (capabilities) of the job prospects while transferring knowledge. In addition, training in the stress-free environment of the clinical skills center enables coaches to allow students to obtain theoretical foundations and practical application of knowledge and problem-solving skills more. In this regard, it is recommended by researchers to use integrated teaching practices in the clinical training planning in order to enhance the clinical performance of students.

## Abbreviations

OSCE: Objective Structured Clinical Examination
CCTST: California Critical Thinking Skills Test
KR-20: Kuder-Richardson formula 20
